# Effectiveness of Vaccines and Antiviral Drugs in Preventing Severe and Fatal COVID-19, Hong Kong

**DOI:** 10.3201/eid3001.230414

**Published:** 2024-01

**Authors:** Yue Yat Harrison Cheung, Eric Ho Yin Lau, Guosheng Yin, Yun Lin, Benjamin J. Cowling, Kwok Fai Lam

**Affiliations:** The University of Hong Kong, Hong Kong, China (Y.Y.H. Cheung, E.H.Y. Lau, G. Yin, Y. Lin, B.J. Cowling, K.F. Lam);; Hong Kong Science and Technology Park, Hong Kong (E.H.Y. Lau, B.J. Cowling);; Imperial College London, London, UK (G. Yin);; Duke-NUS Medical School, Singapore (K.F. Lam)

**Keywords:** molnupiravir, nirmatrelvir, ritonavir, CoronaVac, Comirnaty, COVID-19, respiratory infections, severe acute respiratory syndrome coronavirus 2, SARS-CoV-2, SARS, coronavirus disease, zoonoses, viruses, coronavirus, Hong Kong

## Abstract

We compared the effectiveness and interactions of molnupiravir and nirmatrelvir/ritonavir and 2 vaccines, CoronaVac and Comirnaty, in a large population of inpatients with COVID-19 in Hong Kong. Both the oral antiviral drugs and vaccines were associated with lower risks for all-cause mortality and progression to serious/critical/fatal conditions (study outcomes). No significant interaction effects were observed between the antiviral drugs and vaccinations; their joint effects were additive. If antiviral drugs were prescribed within 5 days of confirmed COVID-19 diagnosis, usage was associated with lower risks for the target outcomes for patients >60, but not <60, years of age; no significant clinical benefit was found if prescribed beyond 5 days. Among patients >80 years of age, 3–4 doses of Comirnaty vaccine were associated with significantly lower risks for target outcomes. Policies should encourage COVID-19 vaccination, and oral antivirals should be made accessible to infected persons within 5 days of confirmed diagnosis.

Since the outbreak of the COVID-19 pandemic in 2019, scientists around the world have raced to discover effective treatments and vaccinations that mitigate the spread of this highly contagious disease; many old drugs have been repurposed for COVID-19 treatment (*1*). Molnupiravir, approved by the US Food and Drug Administration (*2*) for medical use in December 2021, is one of the first oral antiviral drugs shown to inhibit the replication of SARS-CoV-2 virus and to be effective in treating COVID-19 patients (*3*). A double-blind, randomized, controlled, phase 2 trial of unvaccinated and vaccinated patients with early SARS-CoV-2 infection was conducted in the United Kingdom during November 18, 2020–March 16, 2022; results showed molnupiravir recipients had faster median time from randomization to a negative SARS-CoV-2 PCR (primary outcome) than nonrecipients (*4*). Results from MOVe-OUT and MOVe-IN trials suggest that molnupiravir is most effective when treatment is initiated early to patients with mild to moderate COVID-19 who do not require hospitalization but have high risk for severe disease (*5*,*6*). The oral nirmatrelvir/ritonavir combination, also approved by the US Food and Drug Adminstration for medical use in December 2021 (*7*), is another weapon in the arsenal against COVID-19. MOVe-IN, MOVe-OUT, and EPIC-HR trials evaluating nirmatrelvir/ritonavir efficacy suggest that the combination can effectively reduce risks for death and progression to severe disease for patients with mild to moderate COVID-19 (*8*,*9*).

Vaccination is another available tool to mitigate the spread of COVID-19. CoronaVac (Sinovac, http://www.sinovac.com), a whole inactivated virus vaccine, is a popular vaccine choice in the pan-Pacific region and in many developing countries. A double-blind, randomized, controlled, phase 3 trial conducted in Turkey showed CoronaVac has a good safety profile and can effectively reduce the risk for PCR-confirmed, symptomatic SARS-CoV-2 infection and severe COVID-19 (*10*). Results from a large-scale, prospective cohort study in Chile suggested that CoronaVac can effectively prevent SARS-CoV-2 infection and reduce the risks of COVID-19–induced hospitalization, severe disease, and death. Whereas early clinical trials demonstrated the safety and tolerability of CoronaVac among the elderly (*11*), children (*12*), and persons with autoimmune rheumatic diseases (*13*), recent trials have compared the safety and efficacy of the CoronaVac booster with other vaccines (*14*,*15*). Comirnaty (Pfizer-BioNTech, https://www.pfizer.com), an mRNA-based vaccine, is another popular vaccination choice to prevent COVID-19. Similar to the case for CoronaVac, abundant clinical trials and studies have proven safety and efficacy of the Comirnaty vaccine against COVID-19–related hospitalization, death, and severe outcomes (*16*–*19*).

Although clinical trials and studies have demonstrated the efficacy of oral antiviral drugs and vaccinations against COVID-19, real-world evidence is needed to determine the effectiveness of such interventions when used in combination. Furthermore, it would be of interest to explore and evaluate potential interactions, if any, between oral antiviral drugs, vaccinations, and age. Therefore, we compared the effectiveness and interactions of molnupiravir and nirmatrelvir/ritonavir and 2 vaccines, CoronaVac and Comirnaty, in a large population of inpatients with COVID-19.

## Methods

### Study Design

We analyzed data from a territory-wide population cohort of adult hospital inpatients in Hong Kong who had confirmed diagnoses of SARS-CoV-2 infection during March 16–October 31, 2022. Our study received ethics approval from the Institutional Review Board of The University of Hong Kong.

### Data Sources and Study Population

We used data from electronic health records of hospital patients with SARS-CoV-2 infections extracted from the Clinical Management System of Hong Kong Hospital Authority, which is the statutory body that manages all public hospitals in Hong Kong. The deidentified records contained patient age (which we stratified into groups 18–59, 60–79, and >80 years) (*20*–*22*), gender, occupation, symptomatic status, chronic disease history, dates of confirmed SARS-CoV-2 infection, symptom onset, admission date, discharge date, whether infection developed into serious/critical/fatal conditions, and oral antiviral drug prescriptions. We matched and merged patient records with anonymized population-based vaccination records provided by the Centre for Health Protection, Hong Kong Department of Health, via a unique identification key. Vaccination variables were vaccination type, vaccination date, and number of doses. License restrictions apply to the availability of the research data used in this study.

We included adult inpatients if they had a confirmed diagnosis of SARS-CoV-2 infection and were admitted to a local public hospital during the study period. We excluded patients who received both types of oral antiviral drugs (1,421 patients received both molnupiravir and nirmatrelvir/ritonavir), received both types of vaccinations (1,540 patients received both CoronaVac and Comirnaty) or nonlocal vaccinations (167 patients), had missing vaccination dates (49 patients), or received oral antiviral drugs during or after infection developed into a serious or critical condition (362 patients). We evaluated the study population according to treatment and vaccination status (Appendix Table 1).

### Time-Dependent Variable

We indexed the hazard function according to calendar day; oral treatment status was time-dependent. The use of time-dependent treatment status can address the issue of immortal time bias, which arises in many retrospective studies when determining a patient’s treatment status involves a waiting period (e.g., waiting for a prescription to be dispensed). During this waiting period, treated patients are considered immortal because they have to survive until the treatment definition is fulfilled (*23*). To address the issue of immortal time bias, patients who lived long enough to receive treatment were defined as unexposed to the treatment before prescription and only defined as exposed to the treatment on the prescription day and thereafter. When compared with patients who were too ill and did not live long enough to receive the oral treatment, an unexposed treatment status helped resolve the issue of immortal time interval between infection and treatment prescription. If an oral antiviral drug was prescribed, we further indicated whether the prescription was made within or after 5 days of the confirmed COVID-19 diagnosis (*24*–*27*).

We also considered age, gender, Charlson comorbidity index, vaccination type, and number of doses as predictors in our analyses. For generalized-likelihood ratio tests, we evaluated those variables with the first and second order interaction effects between age, vaccination, and oral treatment status.

### Outcomes and Follow-Up Period

The primary outcome was all-cause mortality. The secondary outcome was disease development into a serious/critical/fatal case, comprising a composite outcome of disease progression (all-cause mortality, 3 L/min oxygen supplementation required, intensive care unit admission, intubation, extracorporeal membrane oxygenation, or shock).

The follow-up period for each patient was 28 days, starting from the date of confirmed COVID-19 diagnosis (*28*). We censored data from hospitalized patients who were discharged or never experienced the events of interest within the follow-up period. In the analyses related to the secondary outcome, we defined the date of the event as the date the illness turned serious or critical or the date of in-hospital death, whichever came first.

### Statistical Analysis

We used a proportional hazards regression model with a calendar day setting (*29*–*32*) and time-dependent variables to estimate and compare the effectiveness of molnupiravir and nirmatrelvir/ritonavir treatments and CoronaVac or Comirnaty vaccines against death or progression to severe COVID-19 among hospitalized patients. The hazard function of a patient on calendar day *t* was defined as the baseline hazard function of day *t* multiplied by a function of a predictor, such as age, sex, vaccination status, time-dependent oral treatment status, or their interactions. Because the hazard function was indexed by calendar day, patients were only compared if they had a confirmed COVID-19 diagnosis and were hospitalized during roughly the same period (i.e., with overlapping follow-up windows). Thus, confounding factors arising from the ever-changing baseline hazard function during different waves and periods of the COVID-19 pandemic could then be addressed.

We used the partial-likelihood estimation method to estimate the coefficients of the predictors; each calendar day contributed to 1 term in the log-likelihood function. We adopted the Breslow estimator to estimate the conditional likelihood for the days that had >1 event (i.e., tied observations) and applied the large sample theory to produce approximate variance-covariance matrices for the estimated coefficients.

We initially fitted a full model with all marginal effects and first and second order interaction effects between age, oral treatment status, vaccination type and number of doses. We conducted generalized-likelihood ratio (GLR) tests to analyze the first and second order interaction effects. According to GLR test results, we fitted a reduced model with all marginal effects and significant interactions. We conducted GLR tests to analyze the interaction effects between oral antiviral drugs and vaccinations, between age and oral antiviral drugs, and between age and vaccinations; we then performed subgroup analyses. For each age group, we ran a reduced model with marginal effects only and included the variables age, gender, vaccination type and number of doses, the time-dependent oral treatment status (and whether the treatment was prescribed within 5 days of confirmed diagnosis), and the Charlson comorbidity index. We performed Z-tests for the difference in population means to compare the relative size effects of different oral antiviral drugs, different vaccinations, the same type of vaccination with different numbers of doses, and effects between the antiviral drugs and vaccinations.

We performed all statistical tests and analyses by using RStudio (https://www.rstudio.com) in R version 4.2.1 (The R Project for Statistical Computing, https://www.r-project.org). All statistical tests were 2-sided; we considered p<0.05 statistically significant.

## Results

We identified a total of 39,627 hospitalized adults who had a confirmed diagnosis of SARS-CoV-2 infection during March 16–October 31, 2022, of whom 9,616 received molnupiravir and 10,873 received nirmatrelvir/ritonavir during their hospital stays. Among the 20,489 patients prescribed oral antiviral drugs, 8,708 were >80, 8,996 were 60–79, and 2,785 were <60 years of age. A total of 10,291 hospitalized patients were unvaccinated at the time of confirmed diagnosis, 10,346 had received 1–2 doses of CoronaVac vaccine, 10,166 had received 3–4 doses of CoronaVac vaccine, 576 had received 1 dose of Comirnaty vaccine, and 8,248 had received 2–4 doses of Comirnaty vaccine (Appendix Table 1). Patients were observed during the 28-day follow-up period if they were not discharged or did not experience the outcomes of interest (death or progression to serious illness). 

The cumulative incidences of all-cause mortality were 847 for molnupiravir users, 245 for nirmatrelvir/ritonavir users, and 1,987 for those who did not receive antiviral drugs (controls); cumulative incidences of progression to a serious/critical/fatal case were 1,076 for molnupiravir users, 407 for nirmatrelvir/ritonavir users, and 2,052 for controls (Appendix Table 2). The cumulative incidences of all-cause mortality were 959 for patients partially vaccinated with CoronaVac, 291 for those fully vaccinated with CoronaVac, 51 for those partially vaccinated with Comirnaty, 180 for those fully vaccinated with Comirnaty, and 1,598 for unvaccinated patients; cumulative incidences of progression to a serious/critical/fatal case were 1,104 for those partially vaccinated with CoronaVac, 447 for those fully vaccinated with CoronaVac, 56 for those partially vaccinated with Comirnaty, 248 for those fully vaccinated with Comirnaty, and 1,680 for unvaccinated patients (Appendix Table 3). We defined fully vaccinated status as having >3 doses of CoronaVac and >2 doses of Comirnaty vaccines.

The second-order interaction effects among age, oral antivirals, and vaccinations were not significant for all-cause mortality (p = 0.604) and for progression to serious illness (p = 0.584). Furthermore, the interaction effects between oral antiviral drugs and vaccinations were not significant for all-cause mortality (p = 0.280) and for progression to serious illness (p = 0.341) (Appendix Table 4). The joint effects of oral antiviral drugs and vaccinations were additive. For both target outcomes, significant (or moderate) interaction effects were found between age and oral antiviral drugs and between age and vaccinations (p<0.05) (Appendix Table 4).

Receipt of oral antiviral drugs within 5 days of confirmed COVID-19 diagnosis was associated with significantly lower risk for all-cause mortality in patients >60 years of age (Appendix Table 5). For molnupiravir, the hazard ratios (HRs) for all-cause mortality were 0.65 (95% CI 0.55–0.78) for the 60–79-year age group and 0.61 (95% CI 0.55–0.67) for the >80-year group. For nirmatrelvir/ritonavir, HRs for all-cause mortality were 0.38 (95% CI 0.29–0.49) for the 60–79-year age group and 0.31 (95% CI 0.26–0.36) for the >80-year group. Lower risk for progression to a serious/critical/fatal condition was also observed with antiviral treatments; for molnupiravir, HRs were 0.78 (95% CI 0.67–0.91) for the 60–79-year age group and 0.73 (95% CI 0.67–0.81) for the >80-year group; for nirmatrelvir-ritonavir, HRs were 0.55 (95% CI 0.45–0.67) for the 60–79-year age group and 0.44 (95% CI 0.38–0.51) for the >80-year group. For both age groups, receipt of nirmatrelvir/ritonavir was associated with lower risks than molnupiravir for all-cause mortality and progression to a serious/critical/fatal conditions (p<0.001). No significant clinical benefit was found if the antiviral drugs were prescribed beyond 5 days of confirmed diagnosis or for patients who were <60 years of age, as the corresponding CIs for the HRs contained the value of 1 (Appendix Table 5).

Among patients >60 years of age, receipt of CoronaVac or Comirnaty vaccines was generally associated with lower risks for all-cause mortality and progression to a serious/critical/fatal condition; a greater number of vaccine doses was associated with lower risks. HRs for all-cause mortality in the 60–79-year age group were 0.70 (95% CI 0.56–0.88) for 1 dose of CoronaVac, 0.58 (95% CI 0.47–0.71) for 2 doses, 0.32 (95% CI 0.24–0.42) for 3 doses, and 0.12 (95% CI 0.04–0.37) for 4 doses. HRs for all-cause mortality in the >80-year age group were 0.91 (95% CI 0.81–1.02) for 1 dose of CoronaVac, 0.73 (95% CI 0.64–0.83) for 2 doses, 0.57 (95% CI 0.48–0.69) for 3 doses, and 0.35 (95% CI 0.18–0.69) for 4 doses. For those receiving the Comirnaty vaccine, HRs for all-cause mortality in the 60–79-year age group were 0.70 (95% CI 0.44–1.11) for 1 dose, 0.66 (95% CI 0.50–0.87) for 2 doses, 0.28 (95% CI 0.18–0.43) for 3 doses, and 0.09 (95% CI 0.01–0.65) for 4 doses; in the >80-year age group, HRs were 1.26 (95% CI 0.87–1.81) for 1 dose, 0.56 (95% CI 0.42–0.76) for 2 doses, 0.37 (95% CI 0.25–0.55) for 3 doses, and 0.28 (95% CI 0.07–1.11) for 4 doses. For patients 18–59 years of age, receipt of 3 doses of CoronaVac or Comirnaty was associated with lower risks of all-cause mortality (CoronaVac, HR 0.36 [95% CI 0.19–0.71]; Comirnaty, HR 0.18 [95% CI 0.07–0.45]) and progression to a serious/critical/fatal condition (CoronaVac, HR 0.54 [95% CI 0.34–0.85]; Comirnaty, HR 0.29 [95% CI 0.16–0.51]), whereas no significant benefit was observed for 1 or 2 doses.

Comparisons between CoronaVac and Comirnaty vaccinations showed no significant differences in risks for all-cause mortality or progression to a serious/critical/fatal condition when patients received 1 or 2 doses of either vaccine. For the >80-year age group, those who received 3 doses of Comirnaty vaccine had significantly lower risks for all-cause mortality (p = 0.038) and progression to a serious/critical/fatal condition (p = 0.026) than those who received 3 doses of CoronaVac vaccine.

**Figure 1:**
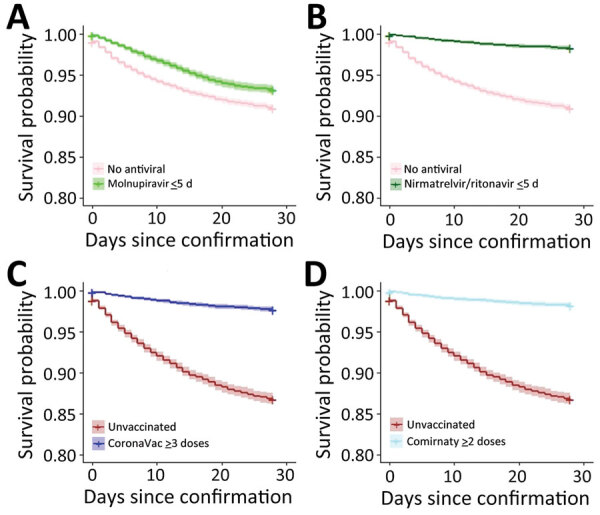
Survival curves for all-cause mortality outcome in study of effectiveness of vaccines and antiviral drugs in preventing severe and fatal COVID-19, Hong Kong. Survival curves were generated to compare patients who did not receive antiviral drugs with those prescribed molnupiravir (A) or nirmatrelvir/ritonavir (B) within 5 days after confirmation of COVID-19 diagnosis and to compare unvaccinated patients with those vaccinated with CoronaVac (C) or Comirnaty (D) vaccines.

**Figure 2:**
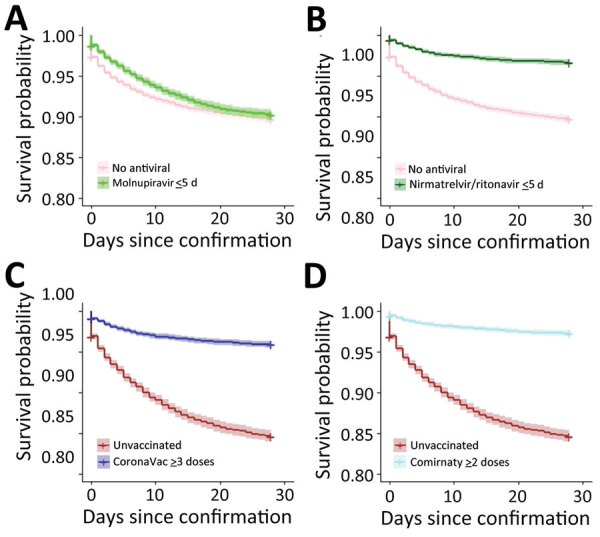
Survival curves for progression to serious/critical/fatal illness outcome in study of effectiveness of vaccines and antiviral drugs in preventing severe and fatal COVID-19, Hong Kong. Survival curves were generated to compare patients who did not receive antiviral drugs with those prescribed molnupiravir (A) or nirmatrelvir/ritonavir (B) within 5 days after confirmation of COVID-19 diagnosis and to compare unvaccinated patients with those vaccinated with CoronaVac (C) or Comirnaty (D) vaccines.

**Figure 3:**
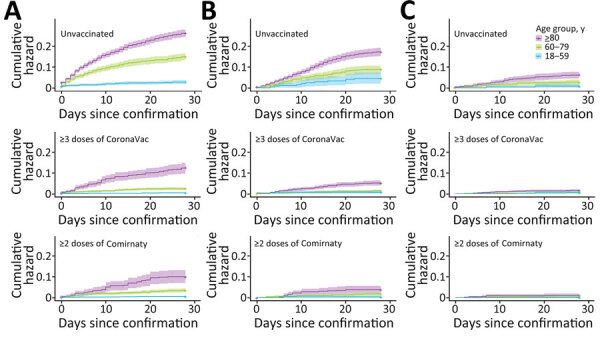
Cumulative hazards for all-cause mortality outcome events in study of effectiveness of vaccines and antiviral drugs in preventing severe and fatal COVID-19, Hong Kong. Cumulative hazards were compared among age groups, patients prescribed oral antiviral drugs, and those unvaccinated or vaccinated with CoronaVac or Comirnaty vaccines. A) No antiviral drugs, B) molnupiravir, C) nirmatrelvir/ritonavir. Antiviral drugs were prescribed within 5 days after confirmation of a COVID-19 diagnosis. Colors indicate age groups within each treatment group.

**Figure 4:**
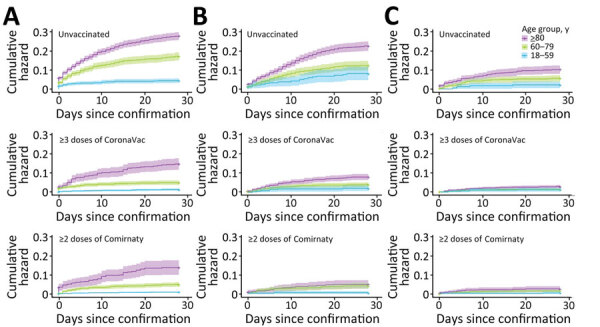
Cumulative hazards for serious/critical/fatal condition outcome events in study of effectiveness of vaccines and antiviral drugs in preventing severe and fatal COVID-19, Hong Kong. Cumulative hazards were compared among age groups, patients prescribed oral antiviral drugs, and those unvaccinated or vaccinated with CoronaVac or Comirnaty vaccines. A) No antiviral drugs, B) molnupiravir, C) nirmatrelvir/ritonavir. Antiviral drugs were prescribed within 5 days after confirmation of a COVID-19 diagnosis. Colors indicate age groups within each treatment group.

Survival was greater for patients who received oral antiviral drugs (within 5 days of diagnosis) than for those who did not receive antiviral drugs (Figures 1, 2). Survival was also greater for patients who received >3 doses of CoronaVac or >2 doses of Comirnaty vaccines than for those who were unvaccinated. Survival curves showed that both oral antivirals and vaccinations effectively reduced the risk for all-cause mortality. Similarly, cumulative hazards for patients who received oral antiviral drugs or vaccinations were lower than for those who did not receive the antiviral drugs and were unvaccinated (Figures 3, 4). Furthermore, vaccination and use of oral antivirals was more effective for patients >80 years of age than for those in the younger age groups.

## Discussion

We conducted a real-world study to compare the effectiveness of SARS-CoV-2 oral antiviral drugs and vaccinations, to examine their interaction effects in a unified model setting, and to address immortal time bias. Hospitalized COVID-19 patients in Hong Kong who received nirmatrelvir/ritonavir had significantly lower all-cause mortality and disease progression risks than those receiving molnupiravir. Interaction effects between the oral antiviral drugs and vaccinations were not significant. The oral antiviral drugs provided greater clinical benefits to the adult patients >60 years of age than to those <60 years of age. Vaccinations generally provided greater clinical benefits for patients <60 years of age. 

Several observational studies have been performed to examine the effectiveness of oral antiviral drugs in patients with mild to moderate COVID-19. A retrospective cohort study of hospitalized patients with confirmed SARS-CoV-2 diagnoses was conducted during February 26–April 26, 2022; the study used propensity score matching to evaluate the effectiveness of molnupiravir and nirmatrelvir/ritonavir for patients who did not require supplemental oxygen at admission (*33*). Our findings on interaction between age and oral antivirals agreed with that study; however, we did not find a significant interaction effect between oral antiviral drugs and vaccinations, which disagreed with that study. We covered a longer study period, and our study population was more inclusive because of the application of the calendar day setting and time-dependent treatment and outcome variables. Another cohort study of nonhospitalized patients showed molnupiravir and nirmatrelvir/ritonavir were effective for patients with mild to moderate COVID-19 (*34*). Compared with that study, the strength of our study population could be attributed to the inpatient setting where clinical outcomes and procedures were systematically documented, and medication adherence was guaranteed. Our findings offer additional insight into the effectiveness of oral antiviral drugs and vaccinations. A different observational study was performed using inverse probability of treatment weighting to adjust the baseline differences between treatment and control groups (*35*). Unlike that study, our analyses involved vaccination records of patients and examined the effectiveness of oral antiviral drugs and vaccinations in a unified model that addressed immortal time bias.

Despite having some advantages over previous studies, the first limitation of our study is that our analyses were inevitably subject to selection bias; therefore, our inpatient setting suggests that the generalizability of our results to outpatients or other settings is questionable. Second, we did not have compliance data for the oral antiviral drugs, and an intention-to-treat handling of the prescription data could underestimate the effects of the oral treatments. However, inpatients are more likely to have a high level of compliance. Third, we were not able to further differentiate all-cause mortality from deaths directly caused by SARS-CoV-2 as opposed to other causes. Fourth, in our modeling of vaccination effect, we did not consider the elapsed time between doses and the potential waning effect of vaccination. Fifth, the difference in treatment timing could have caused bias in our comparison between the 2 types of treatments (i.e., vaccination and oral antiviral drugs). All vaccinated patients prescribed antiviral drugs were vaccinated before they received the drug. The difference in treatment timing could have caused bias in our comparisons between vaccine and antiviral drug effects, as well as during examination of their interaction effects.

In conclusion, this retrospective study of hospitalized patients with COVID-19 showed that the use of vaccinations and oral antiviral drugs was associated with substantial reductions in risks for all-cause mortality and disease progression. Oral molnupiravir and nirmatrelvir/ritonavir were more effective in reducing the risks of target outcomes for adults >60 than for those <60 years of age. In contrast, vaccinations were relatively more effective in reducing the risks for target outcomes for adults <60 than for those >60 years of age. We did not observe significant interaction effects on target outcomes between the use of vaccinations and oral antiviral drugs, and the joint effects of vaccinations and antivirals were additive. Policies should be introduced to encourage COVID-19 vaccinations; oral antivirals should be the standard care for hospitalized patients who have SARS-CoV-2 infection and should be provided within 5 days of confirmed diagnosis. 

**Appendix.** Additional information for effectiveness of vaccines and antiviral drugs in preventing severe and fatal COVID-19, Hong Kong.
