# Supplementary material for: Effectiveness of Vaccines and Antiviral Drugs in Preventing Severe and Fatal COVID-19, Hong Kong
Source: Emerg Infect Dis. 2024 Jan;30(1):70–8. doi: 10.3201/eid3001.230414 (PMC10756371; doi:10.3201/eid3001.230414)
Supplement: Supplementary file 1 — Appendix. Additional information for effectiveness of vaccines and antiviral drugs in preventing severe and fatal COVID-19, Hong Kong. [file 23-0414-Techapp-s1.pdf]

Article DOI: https://doi.org/10.3201/eid3001.230414

EID cannot ensure accessibility for supplementary materials supplied by authors. Readers who have difficulty accessing supplementary content should contact the authors for assistance.

Effectiveness of Vaccines and Antiviral Drugs in Preventing Severe and Fatal COVID-19, Hong Kong

Appendix

Appendix Table 1. Descriptive statistics of the study population according to treatment*

	Oral antiviral drugs				Vaccinations								
					CoronaVac				Comirnaty				
Study Population	Molnupiravir	Nirmatrelvir/ Ritonavir	None	Total	1 dose	2 doses	3 doses	4 doses	1 dose	2 doses	3 doses	4 doses	Unvaccinated
No. persons	9,616 (24.3)	10,873 (24.1)	19,138 (54.7)	39,627	3,611 (9.1)	6,735 (17.0)	9,191 (23.2)	975 (2.5%)	576 (1.5%)	2,758 (7.0%)	5,081 (12.8%)	409 (1.0%)	10,291 (25.9)
Age, y													
Mean (SD)	76.8 (15.2)	72.9 (14.8)	66.7 (20.9)		79.7 (13.7)	73.5 (16.7)	70.9 (15.8)	74.4 (10.9)	64.3 (20.5)	59.1 (21.1)	56.9 (19.4)	69.5 (12.4)	76.1 (17.8)
18–59	1,173 (12.2)	1612 (14.8)	6,304 (32.9)	9,089	295 (8.2)	1,161 (17.3)	1,969 (21.4)	57 (5.8%)	198 (34.4%)	1,236 (44.8%)	2,572 (50.6%)	57 (13.9%)	1,544 (15.0)
60–79	3,574 (37.2)	5,422 (49.9)	6,396 (33.4)	15,392	1,184 (32.8)	2,703 (40.1)	4,231 (46.0)	610 (62.6%)	221 (38.4%)	986 (35.8%)	1,879 (37.0%)	281 (68.7%)	3,297 (32.0)
≥80	4,869 (50.6)	3,839 (35.3)	6,438 (33.7)	15,146	2,132 (59.0)	2,871 (42.6)	2,991 (32.6)	308 (32.6%)	157 (27.2%)	536 (19.4%)	630 (12.4%)	71 (17.4%)	5,450 (53.0)
Gender													
M	4,879 (50.7)	5,620 (51.7)	9,227 (48.2)	19,726	1,782 (49.3)	3,228 (47.9)	4,752 (51.7)	590 (60.5%)	305 (53.0%)	1,318 (47.8%)	2,543 (50.0%)	282 (68.9%)	4,926 (47.9)
F	4,737 (49.3)	5,253 (48.3)	9,911 (51.8)	19,901	1,829 (50.7)	3,507 (52.1)	4,439 (48.3)	385 (39.5%)	271 (47.0%)	1,440 (52.2%)	2,538 (50.0%)	127 (31.1%)	5,365 (52.1)
From nursing home													
Yes	2,776 (28.9)	968 (8.9)	3,860 (20.2)	7,604	1,586 (43.9)	1,231 (18.3)	918 (10.0)	90 (9.2%)	65 (11.3%)	177 (6.4%)	109 (2.1%)	4 (1.0%)	3,424 (33.3)
No	6,840 (71.1)	9,905 (91.1)	15,278 (79.8)	32,023	2,025 (56.1)	5,504 (91.7)	8,273 (90.0)	885 (90.8%)	511 (88.7%)	2,581 (95.6%)	4,972 (97.9%)	405 (99.0%)	6,867 (66.7)
CCI score													
Mean score (SD)	1.05 (1.60)	0.54 (1.42)	0.82 (1.61)	NA	0.95 (1.55)	0.83 (1.62)	0.58 (1.33)	0.46 (1.24)	1.21 (1.96)	0.86 (1.79)	0.45 (1.26)	0.65 (1.62)	1.08 (1.73)
0	5,023 (52.2)	8,296 (76.3)	12,255 (64.0)	25,574	1,943 (53.8)	4,244 (63.0)	6,671 (72.6)	767 (78.7%)	303 (52.6%)	1,854 (67.2%)	4,074 (80.2%)	307 (75.1%)	5,411 (52.6)
1–4	4,268 (44.4)	2,280 (21.0)	6,192 (32.4)	12,740	1,553 (43.0)	2,247 (33.4)	2,320 (25.2)	190 (19.5%)	237 (41.1%)	774 (28.1%)	902 (17.8%)	86 (21.0%)	4,431 (43.1)

Study Population	Oral antiviral drugs: Molnupiravir	Oral antiviral drugs: Nirmatrelvir/ Ritonavir	Oral antiviral drugs: None	Total	Vaccinations: CoronaVac 1 dose	CoronaVac 2 doses	CoronaVac 3 doses	CoronaVac 4 doses	Comirnaty 1 dose	Comirnaty 2 doses	Comirnaty 3 doses	Comirnaty 4 doses	Unvaccinated
5–6	117 (1.2)	41 (0.4)	179 (0.9)	337	35 (1.0)	52 (0.8)	50 (0.5)	5 (0.5%)	9 (1.6%)	21 (0.7%)	27 (0.5%)	2 (0.5%)	136 (1.3)
7–14	208 (2.2)	256 (2.3)	512 (2.7)	976	80 (2.2)	192 (2.8)	150 (1.7)	13 (1.3%)	27 (4.7%)	109 (4.0%)	78 (1.5%)	14 (3.4%)	313 (3.0)
Fully vaccinated†													
Yes	3,871 (40.3)	6,557 (60.3)	7,986 (41.7)	18,414	NA	NA	NA	NA	NA	NA	NA	NA	NA
No	5,745 (59.7)	4,316 (39.7)	11,152 (58.3)	21,213	NA	NA	NA	NA	NA	NA	NA	NA	NA
Received oral antiviral drug													
Yes	NA	NA	NA	20,489	1,639 (45.4)	3,618 (53.7)	6,076 (66.1)	749 (76.8%)	202 (35.1%)	962 (34.9%)	2,351 (46.3%)	290 (70.9%)	4,602 (44.7)
No	NA	NA	NA	19,138	1,972 (54.6)	3,117 (46.3)	3,115 (33.9)	226 (23.2%)	374 (64.9%)	1,796 (65.1%)	2,730 (53.7%)	119 (29.1%)	5,689 (55.3)

*Values are no. (%) except as indicated. CCI, Charlson Comorbidity Index; NA, not applicable.
†Defined as those who had received ≥2 doses of Comirnaty or 3 doses of CoronaVac.

Appenidx Table 2. Target outcomes for molnupiravir and nirmatrelvir/ritonavir recipients compared with nonrecipients

Outcome	Molnupiravir recipients, n = 9,616: Cumulative incidence of events (%)	Person-days	Crude incidence rate/10,000 person-days (95% CI)	Nirmatrelvir/ritonavir recipients, n = 10,873: Cumulative incidence of events (%)	Person-days	Crude incidence rate/10,000 person-days (95% CI)	Nonrecipients, n = 19,138: Cumulative incidence of events (%)	Person-days	Crude incidence rate/10,000 person days (95% CI)
All-cause mortality	847 (8.8)	141,865	59.70 (55.70–63.71)	245 (2.2)	116,658	21.00 (18.37–23.63)	1,987 (10.3)	240,331	82.68 (79.06–86.30)
Severe conditions	1,076 (11.2)	138,779	77.53 (72.92–82.14)	407 (3.7)	115,228	35.32 (31.89–38.75)	2,052 (10.7)	227,669	90.13 (86.25–94.01)

Appendix Table 3. Target outcomes for Coronavac and Comirnaty vaccine recipients compared with unvaccinated persons*

Outcome	CoronaVac recipients, 1–2 doses, n = 11,659: Incidence (%)†	Person-days	Incidence/ 10,000 person-days‡ (95% CI)	CoronaVac recipients, 3–4 doses, n = 10,341: Incidence (%)†	Person-days	Incidence/ 10,000 person-days‡ (95% CI)	Comirnaty recipients, 1 dose, n = 652: Incidence (%)†	Person -days	Incidence/ 10,000 person-days‡ (95% CI)	Comirnaty recipients, 2–4 doses, n = 8,675: Incidence (%)†	Person-days	Incidence/ 10,000 person-days‡ (95% CI)	Unvaccinated, n = 14,049: Incidence (%)†	Person-days	Incidence/ 10,000 person-days‡ (95% CI)
All-cause mortality	959 (8.2)	142,843	67.14 (62.90–71.37)	291 (2.8)	109,291	26.63 (23.60–29.68)	51 (7.8)	7,665	66.54 (48.34 to 84.74)	180 (2.1)	84,406	21.33 (18.21–24.43)	1598 (11.4)	154,649	103.3 (98.29–108.3)
Severe conditions	1,104 (9.5)	137,608	80.23 (75.51–84.94)	447 (4.3)	107,047	41.76 (37.89–45.62)	56 (8.6)	7,508	74.59 (55.13–84.05)	248 (2.9)	83,050	29.86 (26.15–33.57)	1680 (12.0)	146,463	114.7 (109.2–120.2)

*Fully vaccinated status is defined as having received at least two doses of Comirnaty or three doses of CoronaVac.
†Cumulative incidence of events.
‡Crude incidence rate/10,000 person-days.

Appendix Table 4. Generalized likelihood ratio tests for first-order interaction effects between oral antivirals, vaccinations and age*

	Interactions between oral antiviral drugs and vaccinations			Interactions between age and oral antiviral drugs			Interactions between age and vaccinations		
Target outcome	Test statistic	DF	p value	Test statistic	DF	p value	Test statistic	DF	p value
All-cause mortality	14.34	12	0.28	11.01	4	0.03	38.13	12	0.00
Severe conditions	13.39	12	0.34	13.44	4	0.01	20.00	12	0.07

*DF, degrees of freedom.

Appendix Table 5. Association between confounding variables and target outcomes because of COVID-19 according to age group*

	Hazard ratio for all-cause mortality (95% CI)			Hazard ratio for severe conditions (95% CI)		
Variable	18–59 y, n = 9,089	60–79 y, n = 15,392	>80 y, n = 15,146	18–59 y, n = 9,089	60–79 y, n = 15,392	>80 y, n = 15,146
Nirmatrelvir/Ritonavir within 5 days†	0.59 (0.3–1.17)	0.38 (0.29–0.49)	0.31 (0.26–0.36)	0.75 (0.47–1.2)	0.55 (0.45–0.67)	0.44 (0.38–0.51)
Nirmatrelvir/Ritonavir outside 5 days	5.25 (1.24–22.35)	1.51 (0.71–3.21)	0.55 (0.23–1.33)	3.75 (0.91–15.52)	1.43 (0.68–3.03)	0.81 (0.38–1.71)
Molnupiravir within 5 days†	0.78 (0.49–1.22)	0.65 (0.55–0.78)	0.61 (0.55–0.67)	0.86 (0.61–1.22)	0.78 (0.67–0.91)	0.73 (0.67–0.81)
Molnupiravir outside 5 days	NA‡	1.08 (0.59–1.97)	0.94 (0.7–1.26)	0.92 (0.13–6.71)	1.09 (0.6–1.98)	1.12 (0.84–1.49)
CoronaVac 1 dose vs. unvaccinated	0.50 (0.21–1.17)	0.70 (0.56–0.88)	0.91 (0.81–1.02)	0.84 (0.48–1.48)	0.81 (0.66–0.99)	0.93 (0.83–1.04)
CoronaVac 2 doses vs. unvaccinated	0.64 (0.38–1.1)	0.58 (0.47–0.71)	0.73 (0.64–0.83)	0.62 (0.41–0.95)	0.65 (0.54–0.78)	0.75 (0.66–0.85)
CoronaVac 3 doses vs. unvaccinated	0.36 (0.19–0.71)	0.32 (0.24–0.42)	0.57 (0.48–0.69)	0.54 (0.34–0.85)	0.56 (0.45–0.69)	0.62 (0.52–0.73)
CoronaVac 4 doses vs. unvaccinated	0.84 (0.11–6.45)	0.12 (0.04–0.37)	0.35 (0.18–0.69)	1.01 (0.24–4.25)	0.51 (0.31–0.86)	0.50 (0.3–0.84)
Comirnaty 1 dose vs. unvaccinated	0.34 (0.08–1.41)	0.70 (0.44–1.11)	1.26 (0.87–1.81)	0.12 (0.02–0.84)	0.87 (0.59–1.28)	1.07 (0.74–1.56)
Comirnaty 2 doses vs. unvaccinated	0.70 (0.4–1.22)	0.66 (0.5–0.87)	0.56 (0.42–0.76)	0.48 (0.29–0.8)	0.71 (0.55–0.92)	0.65 (0.5–0.84)
Comirnaty 3 doses vs. unvaccinated	0.18 (0.07–0.45)	0.28 (0.18–0.43)	0.37 (0.25–0.55)	0.29 (0.16–0.51)	0.44 (0.32–0.6)	0.41 (0.28–0.58)
Comirnaty 4 doses vs. unvaccinated	NA‡	0.09 (0.01–0.65)	0.28 (0.07–1.11)	NA‡	0.22 (0.07–0.7)	0.25 (0.06–1.02)
Age	1.04 (1.02–1.07)	1.03 (1.02–1.05)	1.04 (1.04–1.05)	1.03 (1.02–1.05)	1.02 (1.01–1.03)	1.04 (1.03–1.05)
Sex						
M	1.07 (0.73–1.55)	1.41 (1.21–1.65)	1.25 (1.14–1.36)	1.13 (0.85–1.51)	1.39 (1.22–1.59)	1.24 (1.14–1.35)
F	Referent	Referent	Referent	Referent	Referent	Referent
Charlson comorbidity index (1-4) §	5.09 (3.21–8.08)	2.19 (1.85–2.58)	1.34 (1.23–1.46)	3.83 (2.76–5.32)	1.85 (1.61–2.13)	1.28 (1.17–1.39)
Charlson comorbidity index (5-6)	7.70 (3.09–19.15)	4.09 (2.81–5.95)	2.30 (1.69–3.13)	5.29 (2.38–11.76)	2.76 (1.91–4.0)	2.22 (1.64–3.01)
Charlson comorbidity index (7-14)	12.31 (6.89–21.96)	5.12 (3.99–6.58)	1.69 (1.27–2.25)	5.43 (3.24–9.1)	3.62 (2.86–4.58)	1.57 (1.18–2.08)

*Target outcomes are all-cause mortality and progression to severe conditions. NA, not applicable.
†Nirmatrelvir/ritonavir or molnupiravir therapy received within 5 days from confirmed diagnoses versus nonrecipients.
‡Hazard ratio was not estimable because the sample size for the subgroup was small and there was no outcome event for the subgroup.
§Charlson comorbidity index scores 1–4 versus zero score.
